# Sex differences and associations between zinc deficiency and anemia among hospitalized adolescents and young adults with eating disorders

**DOI:** 10.1007/s40519-022-01396-5

**Published:** 2022-05-28

**Authors:** Jason M. Nagata, Paola Bojorquez-Ramirez, Anthony Nguyen, Kyle T. Ganson, Christine M. McDonald, Vanessa I. Machen, Amanda Downey, Sara M. Buckelew, Andrea K. Garber

**Affiliations:** 1 Department of Pediatrics, University of California, 550 16th Street, 4th Floor, Box 0110, San Francisco, CA 94158 USA; 2 Yale School of Public Health, Yale University, New Haven, CT 06510 USA; 3 Factor-Inwentash Faculty of Social Work, University of Toronto, Toronto, ON Canada

**Keywords:** Anemia, Feeding and eating disorders, Female, Male, Zinc

## Abstract

**Purpose:**

To determine sex differences in and associations between zinc deficiency and anemia among adolescents and young adults hospitalized for medical complications of eating disorders.

**Methods:**

We retrospectively reviewed electronic medical records of 601 patients aged 9–25 years admitted to the University of California, San Francisco Eating Disorders Program for medical instability, between May 2012 and August 2020. Descriptive statistics, crude, and adjusted logistic regression models were used to assess the association between zinc deficiency (< 55 mcg/dL) and anemia (< 13.6 g/dL in males [M] and < 11.8 g/dL in females [F]).

**Results:**

A total of 87 males and 450 females met eligibility criteria (age 15.98 ± 2.81, 59.4% anorexia nervosa; admission body mass index 17.49 ± 2.82). In unadjusted comparisons, plasma zinc in males and females were not statistically different (M 64.88 ± 14.89 mcg/dL vs F 63.81 ± 13.96 mcg/dL, *p* = 0.517); moreover, there were no differences in the percentage of males and females with zinc deficiency (M 24.14% vs F 24.89%). However, a greater percentage of males than females were anemic (M 50.00% vs F 17.61%, *p* < 0.001), with similar findings in the subgroup with anorexia nervosa. In logistic regression models stratified by sex and eating disorder diagnosis, zinc deficiency was significantly associated with anemia in males (AOR 3.43, 95% CI 1.16, 10.13), but not females (AOR 1.47, 95% CI 0.86, 2.54).

**Conclusions:**

For the first time, we demonstrate that zinc deficiency is equally severe in males compared to females hospitalized with medical complications from eating disorders, with nearly a quarter of inpatients experiencing zinc deficiency. Anemia is more common in males than females hospitalized with eating disorders.

**Level of evidence:**

Level V: descriptive cross-sectional study.

**Supplementary Information:**

The online version contains supplementary material available at 10.1007/s40519-022-01396-5.

## Introduction

Eating disorders in adolescents and young adults can lead to significant medical consequences and nutritional deficiencies, including zinc deficiency [1–4], due to maladaptive dietary restriction [5, 6]. Low levels of zinc may lead to severe health consequences including impairments in immune function, growth, and reproductive function as well as anemia [4, 7, 8]. Zinc has also been implicated in regulation of neural activities involved with sensation, mood, and behavior [9] and zinc-deficient adolescents with eating disorders may be at greater risk for depression and anxiety through metabolic impairments in limbic regions of the brain where zinc is concentrated [10]. This is particularly problematic during adolescence and young adulthood, which is a critical period of development marked by the highest nutritional requirements of the entire human lifespan [11].

Eating disorders in males are under-recognized and under-researched, despite a growing number of males affected by eating disorders [12, 13]. Prior research evaluating zinc deficiency among patients with eating disorders included predominantly female participants or had small sample sizes [1, 14]. In one prior study of zinc deficiency in adults with eating disorders, over 96% of participants identified as female [1]. Another study of zinc deficiency among children with Avoidant Restrictive Food Intake Disorder (ARFID) was limited to a small sample (11 male and 9 female participants) [14]. Similarly, prior research evaluating anemia among patients with eating disorders included exclusively [15] or predominantly [16] female participants, were not disaggregated by sex [17], or had small sample sizes [18]. Targeted research towards sex differences in nutritional status is crucial and directly informs treatment strategies (e.g., refeeding) and could inform sex-specific clinical practice guidelines [19, 20].

Males may present with different eating behaviors including protein overconsumption instead of absolute dietary restriction and muscle-building supplement use, along with more exercise in pursuit of muscularity-oriented goals [12, 13] which may yield sex-specific differences in nutritional deficiencies, such as zinc. Higher protein intake may benefit zinc and iron status in males, since animal sources are highest in concentration of both of these nutrients and contain the most bioavailable form of iron (heme). In females, iron stores can be depleted among patients who are menstruating [21]. Animal studies have demonstrated that zinc is required for iron metabolism [22] and numerous human studies have established the correlation between low zinc and low hemoglobin [23–27]. Zinc could, therefore, plausibly be used as a marker of iron deficiency anemia. However, this relationship has not yet been examined in adolescents and young adults with eating disorders.

To address this gap in the literature, we sought to determine sex differences in plasma zinc concentrations and anemia in adolescents and young adults hospitalized with eating disorders. We also assessed associations between zinc deficiency and anemia by sex.

## Methods

### Study population

We retrospectively reviewed the electronic medical record (EMR) of 601 patients presenting for inpatient hospitalization due to medical instability to the Eating Disorders Program at the University of California, San Francisco (UCSF) between May 2012 and August 2020 who were aged 9–25 years. A psychologist or psychiatrist made the eating disorder diagnosis for the purposes of clinical care. Although a minority of patients (7.8%) were initially diagnosed using DSM-IV criteria (prior to May 2013), we reviewed their clinical and psychosocial characteristics and recategorized them using DSM-5 criteria. For instance, we reviewed the charts of participants with a DSM-IV diagnosis of Eating Disorder Not Otherwise Specified (EDNOS) and reclassified them into an appropriate DSM-5 eating disorder diagnosis [28].

We used the following inclusion criteria to determine the sample: patients aged 9–25 years with an initial hospitalization for the medical management of an eating disorder between May 22, 2012 and August 31, 2020. Patients with missing plasma zinc concentration measurements were excluded from the study (*n* = 64). The final analytic sample consisted of 537 patients.

### Study design

Sociodemographic data, anthropometric measurements, disease characteristics, and lab data were documented in the EMR for each participant during their hospitalization as part of standard clinical care. We retrospectively reviewed their clinical assessments in the EMR and entered them into the UCSF Eating Disorder Program Medical Database. Body mass index (BMI, kg/m^2^) was calculated using initial weight and height measurements assessed at admission. Height is measured on a wall-mounted stadiometer. Weights are obtained using a standardized protocol in a hospital gown without undergarments on a standing scale prior to any intake in the morning, post-void. Percent median BMI (%mBMI) was calculated using calculated BMI and median BMI for age and sex [29]. Plasma zinc and hemoglobin are collected at the time of admission before any inpatient treatment with medications or nutritional supplements as part of the routine admission protocol. All samples were drawn using a standardized lab protocol and analyzed at a Clinical Laboratory Improvement Amendments (CLIA)-certified laboratory, which follows federal standards and regulations. Plasma zinc was analyzed using inductively coupled plasma mass spectroscopy (ICPMS) [30] and hemoglobin was analyzed using spectrophotometry [31]. All lab tests undergo rigorous validation prior to implementing testing, as well as proficiency testing through the College of American Pathologists (CAP) to compare testing results between laboratories. The UCSF clinical laboratory identifies plasma zinc concentrations less than 55 mcg/dL as low and classifies males with hemoglobin concentrations less than 13.6 g/dL and females with hemoglobin concentrations less than 11.8 g/dL as having anemia. These definitions were used in our study to create zinc deficiency and anemia binary variables. Age was stratified into two categories to distinguish between adolescents (< 18 years) and young adults. The binary age variable was used in logistic regression models.

### Ethics

This retrospective chart review study involving human participants was in accordance with the ethical standards of the institutional and national research committee and with the 1964 Helsinki Declaration and its later amendments or comparable ethical standards. The Institutional Review Board (IRB) of the University of California, San Francisco approved this study (20-30323). A waiver of informed consent was approved given no more than minimal risk to participants, the study will not adversely affect the rights and welfare of participants, the retrospective chart review could not practicably be done without the waiver, and an adequate plan to protect the identifiers from improper use and disclosure.

### Statistical analysis

Data analysis was conducted using Stata 15.1 (StataCorp LP, College Station, TX). Unadjusted differences between males and females in demographic characteristics, BMI variables, plasma zinc, hemoglobin, hematocrit, albumin, and anemia were calculated using independent samples *t* tests, Chi-squared tests, or Fisher’s exact tests. Logistic regression analyses were used to examine the association between zinc deficiency and anemia, adjusting for age and eating disorder diagnosis and stratified by sex. Subgroup analyses were conducted for individuals with anorexia nervosa, as this represented the most common eating disorder diagnosis (supplemental appendix).

## Results

This cross-sectional study included 87 male and 450 female adolescent and young adult participants. Demographic and clinical characteristics by sex are shown in Table 1. The average age was 15.98 years and mean %mBMI was 86.74%. There were no significant sex differences in age, race/ethnicity, %mBMI, or BMI. There were sex differences in hospitalization diagnoses (*p* = 0.001), for instance, 42.53% of males versus 62.67% of females had a diagnosis of anorexia nervosa, the most common diagnosis. Subgroup analyses of individuals with anorexia nervosa are shown in the supplemental appendix.

**Table 1:** Demographic and clinical characteristics of adolescents and young adults hospitalized for eating disorders by sex

Characteristic	Total (*N* = 537)^a^	Sex		*p* value^c^
		Male (*N* = 87)^b^	Female (*N* = 450)^b^	
Age (years)	15.98 ± 2.81	16.23 ± 2.62	15.93 ± 2.84	0.363
Age				0.884
Adolescents (< 18 years)	429 (79.89)	70 (80.46)	359 (79.78)	
Young adults (≥ 18 years)	108 (20.11)	17 (19.54)	91 (20.22)	
Race/ethnicity				0.060
Non-Hispanic White	323 (60.15)	44 (50.57)	279 (62.00)	
Hispanic	87 (16.20)	24 (27.59)	63 (14.00)	
Asian or Native Hawaiian and Other Pacific Islanders	41 (7.64)	5 (5.75)	36 (8.00)	
Multiracial	32 (5.96)	5 (5.75)	27 (6.00)	
Other	27 (5.03)	3 (3.45)	24 (5.33)	
Unknown/declined	17 (3.17)	3 (3.45)	14 (3.11)	
Non-Hispanic Black or African American	10 (1.86)	3 (3.45)	7 (1.56)	
Diagnosis				**0.001**
Anorexia Nervosa	319 (59.40)	37 (42.53)	282 (62.67)	
Unspecified Feeding and Eating Disorder (UFED)	53 (9.87)	8 (9.20)	45 (10.00)	
Other	41 (7.64)	11 (12.64)	30 (6.67)	
Avoidant Restrictive Food Intake Disorder (ARFID)	32 (5.96)	12 (13.79)	20 (4.44)	
Other Specified Feeding and Eating Disorder (OSFED)	85 (15.83)	19 (21.84)	66 (14.67)	
Bulimia Nervosa	6 (1.12)	0	6 (1.33)	
Binge-eating disorder	1 (0.19)	0	1 (0.22)	
% median BMI	86.74 ± 13.60	85.88 ± 14.30	86.91 ± 13.47	0.519
BMI (kg/m^2^)	17.49 ± 2.82	17.61 ± 3.04	17.47 ± 2.78	0.662
Zinc (plasma), mcg/dL (normal range 55–150 mcg/dL)	63.98 ± 14.11	64.88 ± 14.89	63.81 ± 13.96	0.517
Zinc (plasma) (mcg/dL)				
Low (< 55 mcg/dL)	133 (24.77)	21 (24.14)	112 (24.89)	0.882
Hemoglobin, g/dL (normal range M: 13.6–17.5; F: 11.8–15.5)	12.95 ± 1.19	13.68 ± 1.28	12.81 ± 1.12	**< 0.001**
Hematocrit %	38.19 ± 3.22	39.68 ± 3.43	37.91 ± 3.10	**< 0.001**
Anemic (M: < 13.6 g/dL; F: < 11.8 g/dL)	119 (22.67)	41 (50.00)	78 (17.61)	**< 0.001**
Albumin (g/dL)	4.39 ± 1.67	4.36 ± 0.42	4.40 ± 1.80	0.871

Table values are mean ± SD for continuous variables and *n* (column %) for categorical variables

^a^Due to missing data, hemoglobin, hematocrit, and anemic have a sample size of 525 and albumin has a sample size of 517

^b^Percentages may not sum to 100% due to rounding

^c^*p* value is for t test, Fisher's Exact, or Chi-squared test as appropriate for continuous and categorical variables, respectively

There were no significant sex differences in mean plasma zinc concentrations (M 64.88 mcg/dL vs F 63.81 mcg/dL, *p* = 0.517) or in the percentage of males versus females identified as having zinc deficiency (M 24.14% vs F 24.89%, *p* = 0.882), with similar findings among the subsample with anorexia nervosa (M 24.32% vs F 23.40%, *p* = 0.901). However, males had significantly higher hemoglobin (M 13.68 g/dL vs F 12.81 g/dL, *p* < 0.001) and hematocrit (M 39.68 g/dL vs F 37.91 g/dL, *p* < 0.001) concentrations. Moreover, a greater percentage of males than females with eating disorders were identified as having anemia (M 50.00% vs F 17.61%, *p* < 0.001), with similar findings among the subsample with anorexia nervosa (M 55.56% vs F 20.14%, *p* < 0.001).

In logistic regression models stratified by sex and adjusted for age and eating disorder diagnosis, zinc deficiency was significantly associated with anemia in males (AOR 3.43, 95% CI 1.16, 10.13) but not in females (AOR 1.47, 95% CI 0.86, 2.54). In the subsample limited to those with anorexia nervosa, findings in males were attenuated and no longer statistically significant (AOR 1.85, 95% CI 0.38, 8.99) and similar in females (AOR 1.77, 95% CI 0.92, 3.38).

## Discussion

This retrospective chart review found that nearly a quarter of male and female adolescents and young adults requiring inpatient medical stabilization from an eating disorder had plasma zinc deficiency. To our knowledge, this is the first study to report zinc deficiency specifically in a male eating disorder sample and to examine sex differences in plasma zinc concentrations. Although the prevalence of zinc deficiency did not differ according to sex, it was associated with a higher odds of anemia in males but not in females. Further, a higher proportion of males were anemic compared to females. Given the myriad of health consequences related to zinc deficiency including impairments in growth, immune function, and reproductive function [4, 7, 8], clinicians should consider assessing for zinc deficiency in both males and females with medical instability from eating disorders and consider additional screening for anemia in males with zinc deficiency. Despite potential sex differences in eating disorder behaviors (e.g., exercising behaviors, muscularity-oriented eating) [12, 32], males present with equally severe zinc deficiency.

Zinc deficiency was associated with anemia in males but not in females in models adjusting for age and eating disorder diagnosis. As the most common nutrient deficiency worldwide, anemia has received appropriate attention in the literature for its contribution to delayed growth and development, increased susceptibility to infection, pregnancy complications, and cardiac problems [4, 7, 8]. Zinc is necessary for iron metabolism, including intestinal absorption and tissue mobilization. The relationship between zinc and hemoglobin reported in human studies [23–27] supports the use of plasma zinc as a marker for iron deficiency anemia [8]. However, this relationship has not been developed in patients with eating disorders. Both trace minerals are critical for erythropoiesis and may have adverse effects on erythrocyte membranes [33]. In animal studies, zinc supplementation was shown to stimulate erythropoiesis in anemic rats and may contribute to tissue accretion during nutritional rehabilitation [22]. Zinc deficiency may also mediate effects of selenium and other essential minerals on hemoglobin [34].

In the subsample limited to individuals with a diagnosis of anorexia nervosa, we found similar trends in sex differences of zinc deficiency and anemia as with the overall sample. Approximately one quarter of males and females hospitalized with anorexia nervosa presented with zinc deficiency, while a greater proportion of males than females presented with anemia. The association between zinc deficiency and anemia was attenuated and no longer statistically significant among males with anorexia nervosa, although the adjusted odds ratio was greater than one (1.85). Given the relatively smaller number of males with anorexia nervosa (*n* = 37), the subgroup analysis could have been underpowered to detect a difference, or there may not be an association among males with anorexia nervosa.

Although anemia is generally less common in males than females, we found that anemia was more common in males compared to females hospitalized for eating disorders, and among the subsample with anorexia nervosa. Males with eating disorders and anemia should be assessed for zinc deficiency and vice versa. While the association between zinc deficiency and anemia was not statistically significant in females, the odds ratio (1.42) suggests anemia is more likely to be concurrent. Future research should examine the biological mechanisms accounting for sex differences in the relationship between zinc deficiency and anemia.

## Limitations/strengths

Limitations of this study include its retrospective and observational nature, which precludes causal inferences. We did not have a healthy control group, though the main research question focused on sex differences among a clinical sample. Although we adjusted for potential confounders such as age and diagnosis, there is the possibility of additional unmeasured confounders. We did not collect data on iron studies, menstrual status (though this would only apply to females), or micronutrient (e.g., zinc, iron) supplementation prior to hospital admission, which could alter the results of the study. Findings are from a tertiary care hospital in Northern California and may not be generalizable to other populations (generalizability bias). Selection bias is a possibility; however, there were no significant differences in demographic or anthropometric data between those included in the study and the 64 participants excluded due to eligibility criteria except that those excluded had a slightly higher BMI (18.3 vs 17.5 kg/m^2^). Our subgroup analyses of individuals with anorexia nervosa, especially among males (*n* = 37) for adjusted regression analyses, may have been underpowered. Future research with larger samples may be able to determine sex differences and sex-stratified associations within layers of feeding and eating disorder diagnoses. Strengths of this study include a robust participant sample gathered over eight years, whose clinical information was gathered by specialized eating disorder treatment teams. It is noteworthy that over 16% of sample participants were male, representing a significant proportion compared to other studies [1]. To our knowledge, this represents the first study to examine sex differences related to zinc deficiency in eating disorders.

## Conclusions

The vast majority of research on the medical complications of eating disorders has been conducted in exclusively or primarily female samples. Because eating disorders and their medical complications affect both males and females, there is a critical need for studies to examine sex differences in medical complications to inform sex-specific clinical practice guidelines. Clinical guidance from the Society for Adolescent Health and Medicine’s Position Paper on the Medical Management of Restrictive Eating Disorders [29] describes certain aspects of medical management that only applies to females [35], such as the use of amenorrhea as a criterion to assess for bone mineral density [35], although deficits in bone density are equally severe in males as in females with anorexia nervosa [36]. Similarly, this study demonstrates that zinc deficiency is equally severe and anemia is more common in hospitalized males with eating disorders compared to females, and underscores the dire need for further research to develop sex-specific clinical practice guidelines in caring for young people with eating disorders.

### What is already known on the subject?

Prior research evaluating zinc deficiency in eating disorders were predominantly female or had small sample sizes.

### What this study adds?

We demonstrate for the first time that zinc deficiency is equally prevalent in males compared to females with eating disorders, with nearly a quarter of inpatient experiencing zinc deficiency.

## Supplementary Information

Below is the link to the electronic supplementary material.

Supplementary file1 (DOCX 15 KB)

## Data Availability

The data that support the findings of this study are available on request from the corresponding author, JN. The data are not publicly available due to confidentiality restrictions, e.g., their containing information that could compromise the privacy of research participants.
