# Supplementary material for: Sex differences and associations between zinc deficiency and anemia among hospitalized adolescents and young adults with eating disorders
Source: Eat Weight Disord. 2022 May 28;27(7):2911–7. doi: 10.1007/s40519-022-01396-5 (PMC9556365; doi:10.1007/s40519-022-01396-5)
Supplement: Supplementary file 1 — Supplementary file1 (DOCX 15 KB) [file 40519_2022_1396_MOESM1_ESM.docx]

| Supplemental Appendix. Demographic and clinical characteristics of adolescents and young adults hospitalized for anorexia nervosa by sex ^a^ | | | | |
| --- | --- | --- | --- | --- |
|  |  | Sex | |  |
| Characteristic | Total  (N=319)^b^ | Male  (N = 37)^c^ | Female  (N = 282)^c^ | P value ^d^ |
| Age, years | 16.22 ± 2.88 | 16.61 ± 2.39 | 16.17 ± 2.93 | 0.377 |
| Age |  |  |  | 0.883 |
| Adolescents (<18 years) | 247 (77.43) | 29 (78.38) | 218 (77.30) |  |
| Young adults (≥ 18 years) | 72 (22.57) | 8 (21.62) | 64 (22.70) |  |
| Race/ethnicity |  |  |  | 0.258 |
| Non-Hispanic White | 200 (62.70) | 18 (48.65) | 182 (64.54) |  |
| Hispanic | 52 (16.30) | 11 (29.73) | 41 (14.54) |  |
| Asian or Native Hawaiian and Other Pacific Islanders | 27 (8.46) | 3 (8.11) | 24 (8.51) |  |
| Multiracial | 10 (3.13) | 1 (2.70) | 9 (3.19) |  |
| Other | 15 (4.70) | 2 (5.41) | 13 (4.61) |  |
| Unknown/declined | 10 (3.13) | 1 (2.70) | 9 (3.19) |  |
| Non-Hispanic Black or African American | 5 (1.57) | 1 (2.70) | 4 (1.42) |  |
| % median BMI | 83.84 ± 10.96 | 83.52 ± 10.33 | 83.88 ± 11.06 | 0.852 |
| BMI, kg/m^2^ | 16.98 ± 2.34 | 17.36 ± 2.25 | 16.93 ± 2.35 | 0.287 |
| Zinc (plasma), mcg/dL (normal range 55 – 150 mcg/dL) | 64.20 ± 15.15 | 66.77 ± 18.33 | 63.86 ± 14.69 | 0.273 |
| Zinc (plasma), mcg/dL |  |  |  | 0.901 |
| Low (<55 mcg/dL) | 75 (23.51) | 9 (24.32) | 66 (23.40) |  |
| Hemoglobin, g/dL (normal range M: 13.6-17.5; F: 11.8-15.5) | 12.82 ± 1.17 | 13.52 ± 1.22 | 12.73 ± 1.13 | **<0.001** |
| Hematocrit % | 37.77 ± 3.24 | 39.26 ± 3.42 | 37.58 ± 3.17 | **0.003** |
| Anemic (M: <13.6 g/dL; F: <11.8 g/dL) | 76 (24.20) | 20 (55.56) | 56 (20.14) | **<0.001** |
| Albumin, g/dL | 4.31 ± 0.40 | 4.33 ± 0.39 | 4.31 ± 0.40 | 0.792 |
| ^a^ Table values are mean ± SD for continuous variables and n (column %) for categorical variables.  ^b^ Due to missing data, hemoglobin, hematocrit, and anemic have a sample size of 314 and albumin has a sample size of 307. | | | | |
| ^c^ Percentages may not sum to 100% due to rounding. | | | | |
| ^d^ P value is for t test, Fisher's Exact, or Chi-Squared test as appropriate for continuous and categorical variables, respectively. | | | | |
